# Transitioning from Cangrelor to Oral P2Y_12_ Inhibitors in Patients with ACS: Insights from the ARCANGELO Study

**DOI:** 10.2174/0115701611309982250522065849

**Published:** 2025-06-02

**Authors:** Leonardo De Luca, Paolo Calabrò, Piera Capranzano, Elisa Nicolini, Ciro Mauro, Carlo Trani, Francesco Versaci, Fabrizio Tomai, Alessio Mattesini, Martino Pepe, Sergio Berti, Carlo Cernetti, Giuseppe Musumeci, Plinio Cirillo

**Affiliations:** 1 Direttore S.C. Cardiologia, Fondazione I.R.C.C.S. Policlinico San Matteo Viale Camillo Golgi 19, 27100 Pavia, Italy;; 2 UOC Cardiologia Clinica con UTIC. A.O.R.N. Sant'Anna e San Sebastiano, Caserta, Italy;; 3 Cardiology Division, Policlinico Hospital, University of Catania, Catania, Italy;; 4 UO Cardiologia Interventistica, strutturale e pediatrica, Ospedali Riuniti Ancona;; 5 Cardiologia UTIC con emodinamica AORN Cardarelli Napoli, Italy;; 6 "UOC Interventistica Cardiologica e diagnostica invasiva Fondazione Policlinico Universitario A. Gemelli, IRCCS" Roma, Italy;; 7 Department of Systems Medicine, University of Rome Tor Vergata, Roma, Italy;; 8 UOC di Cardiologia Aurelia Hospital, Roma, Italy;; 9 Interventistica Cardiologica Strutturale A.O.U. Careggi, Firenze, Italy;; 10 Cardiologia Universitaria A.O.U. Consorziale Policlinico di Bari, Italy;; 11 Fondazione C.N.R. Reg. Toscana G. Monasterio Pisa, Italy;; 12 Cardiologia Ospedale Ca' Foncello Treviso, Italy;; 13 SC Cardiologia, AO Ordine Mauriziano, Torino, Italy;; 14 Dipartimento di Scienze Biomediche Avanzate, Cardiologia, AOUP "Federico II" Napoli, Italy

**Keywords:** Cangrelor, bleeding, real-world evidence, acute coronary syndrome, P2Y_12_ inhibitor, PCI

## Abstract

**Aims:**

The present analysis of the ARCANGELO study aims to investigate the effect of switching to different oral P2Y_12_ inhibitors when using cangrelor during PCIs in patients with ACS.

**Methods:**

Out of the 995 patients meeting the criteria for this investigation, 138 transitioned to Clopidogrel (CLO), 127 to prasugrel (PRA), and 730 to Ticagrelor (TICA). Compared to the patients on PRA or TICA, users of CLO were older (median (Q1-Q3) 74(64-81) years CLO, 59(54-65) years PRA, 65(56-73) TICA; p<0.0001), had more comorbidities (37.0% CLO, 17.3% PRA, 18.9% TICA, p<0.0001), and had more frequently an NSTEMI diagnosis (68.1% CLO *vs* 33.1% PRA *vs* 35.9% TICA, p<0.0001).

**Results:**

Five moderate bleedings were recorded without any severe episodes. There were no significant differences in the bleeding rate when switching to the different oral P2Y_12_ inhibitors (2.2% CLO, 5.3% TICA, 7.9% PRA, p = 0.0705) while different incidences of MACEs (4.3% CLO, 1.1% TICA, 0% PRA, p = 0.0113) and NACEs (4.3% CLO, 1.8% TICA, 0% PRA, p=0.0321) were observed during the 30 days of the study.

**Conclusion:**

The use of cangrelor and the switch to any oral P2Y_12_ inhibitor in compliance with the EU SmPC is safe, with a low risk of ischemic events in routine clinical practice.

## 1 INTRODUCTION

Treatment of Coronary Artery Disease (CAD) patients with potent P2Y_12_ inhibitors is considered standard therapy because it significantly reduces short- and long-term ischemic events [1, 2]. The delayed onset of action of oral P2Y_12_ inhibitors limits their use for urgent or periprocedural treatment of patients undergoing Percutaneous Coronary Intervention (PCI) [3]. Cangrelor is the only intravenous P2Y_12_ inhibitor currently available. Clinical trials taking to cangrelor registration were performed when Clopidogrel (CLO) was the only oral P2Y_12_ inhibitor available [4-9]. These studies have consistently shown that cangrelor reduces ischemic events during PCI [7]. Limited experiences in the use of cangrelor and transition to oral P2Y_12_ inhibitors are available [10-12]. None of these investigated the differences in the use of the different drugs.

The itAlian pRospective Study on CANGrELOr (ARCANGELO) is a multicenter, observational, prospective cohort Post-Authorization Safety Study (PASS) designed in agreement with the guidance for category 3 [13], performed to assess the safety of cangrelor in a real-world setting and describe bleeding and Major Adverse Cardiac Events (MACEs) rates in patients undergoing PCI that require treatment with IV cangrelor switching to either Prasugrel (PRA), CLO, or Ticagrelor (TICA). The study protocol and the main results from the interim and final analyses have been presented in previous articles [14, 15]. The incidence of bleeding within 30 days post-PCI resulted below the expectations both in the interim and final analyses [14, 15], confirming the satisfactory safety profile of cangrelor when it is used according to the indications for use [16].

This article aims to report the findings of a post-hoc analysis of the ARCANGELO study to highlight variations in patient types and results when transitioning to different oral P2Y_12_ inhibitors while using cangrelor for PCI.

## 2 METHODS

The ARCANGELO study aimed to assess the safety of cangrelor in a real-world setting in Italy when administered in patients with acute coronary syndromes undergoing PCI procedures and in whom oral therapy with P2Y_12_ inhibitors was not feasible or desirable.

The start of the observation period was defined as the start of cangrelor administration. The observation period ended at the last visit, about 30 days after PCI. Upon the investigator’s decision to treat with cangrelor during PCI, patients willing to take part in the study were invited to sign an informed and privacy consent form for inclusion in the study. If patients could not give their informed consent before the PCI because of their medical condition, the investigator collected written informed and privacy consent from the patient after the PCI as soon as the patient’s condition permitted it and before discharge. The study was conducted in 28 Italian high-volume centers with a 24-hour available catheterism lab over 12 months. The total sample was 1004 enrolled patients. CT registration number: NCT04471870. [14].

This post-hoc analysis aimed to investigate the demographic and clinical characteristics of patients transitioning from cangrelor to oral antiplatelet transition therapy with CLO, PRA, or TICA. Patients switched to any of the oral P2Y_12_ inhibitors were also compared by type of intervention and incidence of Bleeding Academic Research Consortium (BARC) bleedings, MACEs, Intraprocedural Thrombotic Events (IPTE), Treatment-Emergent Adverse Events (TEAE), and Net Adverse Clinical Events (NACEs) defined as BARC 3-5 bleedings or MACE events. The timing of the administration of CLO, PRA, or TICA was also assessed.

Since the ARCANGELO study has no inferential aims, statistical tests comparing the differences between transition therapy groups were exploratory. The results of the statistical analyses were summarized by descriptive statistics, including frequency count and percentage for categorical variables, number of observations, mean ± Standard Deviation (SD) or median (Q1-Q3), and continuous variables, depending on data distribution. Statistical tests were performed on the eligible population using the Kruskal-Wallis or Analysis of Variance (ANOVA) test for continuous variables (depending on data distribution) and Bonferroni correction, when appropriate, or the Fisher exact test for categorical variables using the SAS software. Missing data were not replaced.

## 3 RESULTS

A total of 995 of the 1004 patients enrolled in the ARCANGELO study had data on the transition to an oral P2Y_12_ inhibitor and were eligible for this analysis. 138 of them were administered CLO, 127 PRA, and 730 TICA as their first oral P2Y_12_ inhibitor (Fig. **1**). Patients with CLO were older than those with TICA and PRA: the median age was 74 (64-81) years in patients with CLO, 65 (56-73) in patients with TICA, and 59 (54-65) years in those with PRA (p<0.0001). Males were prevalent in each group, ranging from 69.6% in patients with CLO up to 81.9% in patients with PRA (p=0.0420) (Table **1**).

Patients’ distribution by type of ACS was different: more than twice as many patients treated with PRA (64.1%) or TICA (66.9%) had STEMI than those treated with CLO (31.9% - p<0.0001). The percentage of patients with a family history of CAD was higher in patients with TICA (36.1%) than those with CLO (26.8%) and PRA (26.5%) (p=0.0363). The proportion of patients with prior relevant medical conditions was almost double, 37.0% in patients treated with CLO than in those using TICA (18.9%) or PRA (17.3% - p<0.0001). The rate of patients with at least an ongoing comorbidity was 95.7% in patients with CLO, 82.7% with PRA, and 81.0% with TICA (p<0.0001), having a significantly higher frequency of hypertension, diabetes mellitus, and atrial fibrillation (Table **1**).

The mean duration of cangrelor infusion was 151.4 (±43.3) minutes in patients with CLO, 150.0 (±122.7) minutes in patients with TICA, and 140.2 (±34.1) minutes in those with PRA (p=0.6192).

Radial access was the most common catheter access site in each subgroup. Primary PCI duration was statistically different between CLO (52.7 ± 35.9 minutes), PRA (41.4 ± 26.2 minutes), and TICA (48.5 ± 37.3 minutes) treated patients (p=0.0363). Peri-procedural or bailout use of GPIIb/IIIa Inhibitors (GPI) was observed in each group (1.4% in patients with CLO, 2.4% in patients with PRA, and 2.9% in patients with TICA) (Table **2**).

The majority of patients (563 of 900 patients for whom the time to transition was recorded) were treated with a loading dose of an oral P2Y_12_ inhibitor at the end of the cangrelor infusion. However, in 337 patients treated before the end of the cangrelor infusion (290 with TICA, 45 with PRA, and 2 with CLO), the loading dose of these drugs was administered at a median time of 30 minutes before stopping cangrelor (Fig. **2**).

Out of the 730 patients transferred to TICA for which data is available, 723 received the loading dose of 180 mg; 717 (98.2%) of them started the maintenance regimen of 90 mg twice daily. Out of the 138 patients treated with CLO for which data are available, 100 (72.5%) received a loading dose of 600 mg, while the other 34 patients (24.6%) received 300 mg, and 133 (96.4%) of them started the maintenance regimen of 75 mg once daily. All the patients treated with PRA for which data were available, (99.2%) received a loading dose of 60 mg followed by a maintenance dose of 10 mg once daily.

Thirty-six of the 60 patients who changed their P2Y_12_ during the study switched from TICA to CLO, 15 from TICA to PRA, 6 from CLO to TICA, 2 from PRA to CLO, and 1 from PRA to TICA; the main reason for using more than one oral P2Y_12_ inhibitor in these patients was not reported (Table **3**).

Cangrelor has been used off-label in 119 out of 995 patients (12.0%). The most frequent reasons included: TICA or PRA loading dose administered more than 30 minutes before cangrelor infusion end (n=74 and n=4, respectively); TICA loading dose administered before the start of cangrelor infusion (n=16) and cangrelor duration < 2 hours (n=12).

No safety issues were observed in the cases where off-label timing use to transition to TICA or PRA was observed.

In the 48 hours (± 24 hours) following the PCI, the incidence of BARC type 1-2 (mild) bleeding ranged from 1.4% (n=2) in patients with CLO to 6.3% (n=8) in those with PRA (p = 0.0705), while BARC type 3-5 (moderate-severe) bleedings were observed only in 3 (0.4%) patients treated with TICA (p = 1.0000). During the 30 days of the study, the incidence of BARC type 1-2 bleeding ranged from 2.2% (n=3) in patients with CLO to 7.9% (n=10) in those with PRA (p = 0.0994), while BARC type 3-5 (moderate-severe) bleedings were observed in 5 of the patients with TICA (0.7%) (p = 1.0000) (Fig. **3**).

The 3 (0.4%) of the 52 patients experiencing at least one bleeding within the 30 days post-PCI correlated, among other factors, to cangrelor were using TICA. A total of 7 patients experienced a bleeding event requiring transfusion within the 30 days post-PCI: 6 (0.8%) treated with TICA and 1 (0.8%) with PRA (p = 0.6893).

Three (2.2%) patients treated with CLO and 7 (1.0%) with TICA (p = 0.1827) experienced at least one MACE within 48 hours (±24 hours) post-PCI (p = 0.1827); the corresponding incidences at 30 days were 4.3% (n=6) in patients with CLO and 1.1% (n=8) in those with TICA; no MACEs were observed in patients with PRA during the study (n=127) (p = 0.0113). Three of the patients treated with CLO (2.2%) and 3 of those treated with TICA (0.4%) died, while 1 patient treated with CLO (0.7%) and 1 treated with TICA (0.1%) experienced an ST (Table **4**).

The incidence of IPTE within 48 hours post-PCI ranged from 1.2% (n=9) in patients treated with TICA (0.5% no-reflow and 0.3% slow re-flow) to 2.2% (n=3) with CLO. Patients experiencing at least one NACE during the observation period were 13 (1.8%) of those treated with TICA and 6 (4.3%) with CLO; no NACEs were observed in patients with PRA (p=0.0321).

A total of 30 (21.7%) patients treated with CLO, 28 (22%) with PRA, and 168 (23%) with TICA (p = 0.9533) experienced at least one TEAE during the observation period; the corresponding incidence of serious TEAEs were 13 (9.4%) for CLO, 4 (3.1%) for PRA and 36 (4.9%) for TICA (p = 0.0619). TEAEs related to cangrelor were observed in 6 (0.8%) patients treated with TICA (p = 0.6598).

## 4 DISCUSSION

The ARCANGELO study was designed according to stringent requirements of the regulatory authorities and was conducted to evaluate the transition to all P2Y_12_ inhibitors after using cangrelor during PCI. Each patient had to be treated following the most appropriate clinical practice, as per the Investigator’s judgment [14, 15]. This subanalysis evaluated how Italian interventional cardiologists approach the transition from cangrelor to other oral P2Y_12_ inhibitors.

Confirming the outcomes from other observational trials [17], ticagrelor was the oral P2Y_12_ inhibitor more frequently used in PCI-treated patients after cangrelor infusion. Patients who were older and had more comorbidities—such as chronic kidney disease and a history of anemia—that could increase their risk of bleeding were more frequently switched to clopidogrel [18]**.** Furthermore, for patients with atrial fibrillation requiring treatment with low molecular weight heparin, vitamin K antagonist, or DOACs (oral direct factor Xa inhibitors and direct thrombin inhibitors), including [19] Dual Antiplatelet Therapy (DAPT) with aspirin plus clopidogrel, was the treatment of choice. These observations confirm that the Italian investigators participating in the ARCANGELO study are perfectly in line with the current recommendations on the use of DAPT, especially in patients with high bleeding risk [20, 21]. Furthermore, these results provide a snapshot of the real-world approach of the Italian interventional cardiologists in the transition from cangrelor to oral P2Y_12_ inhibitors because more potent P2Y_12_ inhibitors were widely used, and clopidogrel was preferred for transition in patients for whom the perception of an increased bleeding risk was higher. The correctness of this type of approach is demonstrated by the data on bleeding in the present study, since the incidence of bleeding was 2.2% with clopidogrel, 5.3% with ticagrelor, and 7.9% with prasugrel, even if clopidogrel was most frequently used in the older patients included in the ARCANGELO study [22]. Interestingly, the rate of bleeding events observed in the present report is significantly lower than that observed in the studies belonging to the CHAMPION program (16.8%). In those studies, clopidogrel was the P2Y_12_ inhibitor of choice for transition [5, 6, 7, 9, 23]. Thus, it might be speculated that, in our real-world experience, however, more potent P2Y_12_ inhibitors were widely administered for transition after cangrelor infusion during PCI. A more accurate clinical evaluation of the bleeding risk might have driven a more appropriate antiplatelet therapy to reduce the incidence of bleeding events. Two other real-world registries by the same research group have recently evaluated bleeding after the transition to P2Y_12_ inhibitors other than clopidogrel in cangrelor-treated patients. However, one of them was focused on patients with cardiogenic shock as a clinical presentation only. Moreover, since both studies were published as research letters, they enrolled a small number of patients. They imported a limited number of mild to moderate bleeding events without explaining the actual rate of these events according to the P2Y_12_ inhibitor used, as we do in the present report [24, 25].

Another possible explanation for the lower rate of bleeding observed as compared to the CHAMPION trials is that interventional cardiologists preferred radial access to perform coronary revascularization in our study, while femoral access was widely used in those trials [26]. One of the aspects of bleeding avoidance strategies in PCI includes a selection of vascular access, as well as antithrombotic treatment regimens [27]. We might speculate that both these aspects have significantly improved in PCI management in recent times, finally contributing to the reduced bleeding complications observed.

Transition to oral P2Y_12_ inhibitors has been recently investigated in the CAMEO Registry, [17] indicating a significant interhospital variability in cangrelor dosing and administration of oral P2Y_12_ inhibitors. Specifically, in that study, only a quarter of patients were treated consistently with trial‐established, FDA‐approved recommendations. Interestingly, MACEs occurred in 10.3% of patients not treated according to the established treatment strategy compared to 6.3% of the same events observed in patients treated according to the established treatment strategy. This study's overall rate of MACEs was significantly lower than that of the CAMEO. Moreover, we have observed, for the first time, the rate of MACEs in patients who were treated in line to cangrelor SmPC indications for transition accordingly to the different P2Y_12_ inhibitors: clopidogrel (4.3%), prasugrel (0%), and ticagrelor (0.1%). A possible explanation for these different results might be that, in the CAMEO registry, a longer time gap occurred between cangrelor discontinuation and P2Y_12_ inhibitor administration, thus permitting a vulnerable period of “interruption” in antiplatelet therapy. About 50% of patients who transitioned to clopidogrel had a >1‐hour gap, while this occurred less frequently with prasugrel and with ticagrelor. This time gap was not observed in our study since the transition timing was done according to SmPC indications.

As with other post-hoc analyses, these observational ARCANGELO study results must be interpreted cautiously. Even if we have used the Bonferroni correction when comparing multiple groups to mitigate the risk of Type I errors, the findings from this post-hoc analysis should be considered exploratory and need to be validated with further research. Furthermore, the presented analyses were performed without any adjustment or inclusion of any covariate because of the low frequency of the examined events in each of the samples; thus, all the presented comparisons have only an indicative, non-inferential value. Additionally, because of the few events (bleedings, MACEs, NACEs) observed in the study, any model to identify their predicting factors was not applicable from a statistical standpoint.

## CONCLUSION

This post-hoc analysis of a real-world study confirms that cangrelor is safe. Moreover, these data suggest that the transition from cangrelor to oral P2Y_12_ inhibitors can be safely performed, with an accurate clinical evaluation of the patient ischemic and bleeding risk. Timing of the transition might play a pivotal role in reducing the risk of potential ischemic risk due to partial interruption of antiplatelet therapy. Although randomized studies should be performed to have a clearer point of view on this important topic of antithrombotic therapy, these results might be helpful in daily practices in indicating how to perform the transition from cangrelor to the different oral P2Y_12_ inhibitors.

## Figures and Tables

**Fig. (1):**
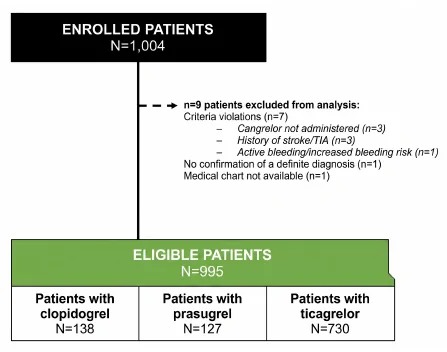
Patient workflow by type of oral P2Y_12_ inhibitor used.

**Fig. (2):**
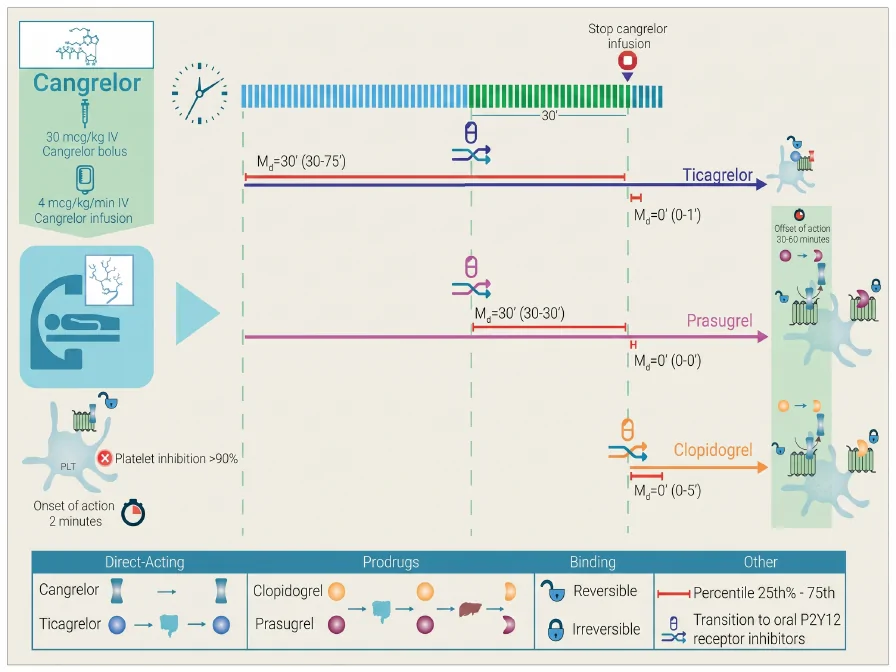
Transition to the different oral P2Y_12_ inhibitors - Timing and mechanisms of action.

**Fig. (3):**
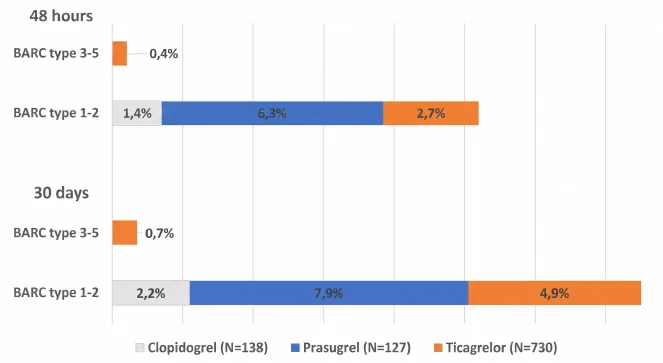
Percentage of patients with bleeding by oral P2Y_12_ inhibitor. **Legend**: BARC 1-2: Bleeding Academic Research Consortium grade 1-2 bleeding; BARC 3-5: Bleeding Academic Research Consortium grade 3-5 bleeding.

**Table 1:** Patient's demographic and clinical history by oral P2Y_12_ inhibitor used.

-	Clopidogrel (CLO) N=138	Prasugrel (PRA) N=127	Ticagrelor (TICA) N=730	Eligible Patients N=995	P
**Demographic Characteristics**					
**Age (years)^§^**	74 (64-81)	59 (54-65)	65 (56-73)	65 (56-73)	<0.0001
**Age ≥ 75 years**	65 (47.1%)	4 (3.1%)	146 (20.0%)	215 (21.6%)	<0.0001
**Female**	42 (30.4%)	23 (18.1%)	159 (21.8%)	224 (22.5%)	0.0420
**Weight < 60 kg**	11 (8.0%)	3 (2.4%)	37 (5.1%)	51 (5.1%)	0.1302
BMI (kg/m^2^)*	27.0 (4.1)	28.2 (4.2)	27.5 (4.3)	27.5 (4.3)	0.0834
Systolic BP (mmHg)*	134.84 (25.50)	137.20 (26.77)	137.30 (24.48)	136.94 (24.91)	0.5758
Diastolic BP (mmHg)*	74.10 (14.08)	80.98 (14.36)	79.27 (14.10)	78.74 (14.25)	0.0001
Heart rate (bpm)*	73.15 (21.54)	75.13 (16.94)	73.67 (17.94)	73.78 (18.36)	0.6666
**Current smoker**	33 (24.8%)	64 (51.2%)	265 (37.6%)	362 (37.6%)	<0.0001
**Clinical History**					
**STEMI**	44 (31.9%)	85 (66.9%)	468 (64.1%)	597 (60.0%)	<0.0001
**NSTE-ACS**	94 (68.1%)	42 (33.1%)	262 (35.9%)	398 (40.0%)	
**NSTEMI**	47 ( 34.1%)	34 ( 26.8%)	191 ( 26.2%)	272 ( 27.3%)	-
**UA**	47 ( 34.1%)	8 ( 6.3%)	71 ( 9.7%)	126 ( 12.7%)	-
**Multi-vessel coronaropathy**	72 (52.2%)	68 (53.5%)	369 (50.5%)	509 (51.2%)	0.7950
**2 vessels^**	40 (55.6%)	44 (64.7%)	218 (59.1%)	302 (59.3%)	0.5398
**≥ 3 vessels^**	32 (44.5%)	24 (35.3%)	151 (40.9%)	207 (40.7%)	
**LAD**	98 ( 71.0%)	89 ( 70.1%)	510 ( 69.9%)	697 ( 70.1%)	0.897
**CX**	54 (39.1%)	56 (44.1%)	305 (41.8%)	415 (41.7%)	0.7165
**RCA**	78 (56.5%)	67 (52.8%)	376 (51.5%)	521 (52.4%)	0.5519
**LMCA**	11 (8.0%)	6 (4.7%)	48 (6.6%)	65 (6.5%)	0.6029
**Family history of CAD**	30 (26.8%)	31 (26.5%)	217 (36.1%)	278 (33.5%)	0.0363
**Prior relevant medical conditions**	51 (37.0%)	22 (17.3%)	138 (18.9%)	211 (21.2%)	<0.0001
**Comorbidities**	132 (95.7%)	105 (82.7%)	591 (81.0%)	828 (83.2%)	<0.0001
**Hypertension**	115 (83.3%)	83 (65.4%)	459 (62.9%)	657 (66.0%)	<0.0001
**Hyperlipidaemia**	77 (55.8%)	56 (44.1%)	336 (46.0%)	469 (47.1%)	0.0825
**Diabetes mellitus**	41 (29.7%)	22 (17.3%)	143 (19.6%)	206 (20.7%)	0.0198
**Atrial fibrillation**	31 (22.5%)	0 (0.0%)	5 (0.7%)	36 (3.6%)	<0.0001
**COPD**	12 (8.7%)	4 (3.1%)	28 (3.8%)	44 (4.4%)	0.0494
**PAD**	10 (7.2%)	3 (2.4%)	25 (3.4%)	38 (3.8%)	0.0889
**Chronic kidney disease**	7 (5.1%)	1 (0.8%)	15 (2.1%)	23 (2.3%)	0.0638
**Anaemia**	3 (2.2%)	1 (0.8%)	7 (1.0%)	11 (1.1%)	0.3581
**Renal failure**	2 (1.4%)	0 (0.0%)	5 (0.7%)	7 (0.7%)	0.4363

**Note:** Values are mean (SD); median (Q1-Q3); n patients (%).

Fisher exact test for dicotomic variables.

^ Percentages were calculated on eligible patients with multi-vessel as type of coronaropathy

* mean (SD); parametric ANOVA test.

^§^ median (Q1-Q3); Kruskal-Wallis test.

Legend: bpm=beats per minute; BMI=body mass index; BP=blood pressure; COPD= chronic obstructive pulmonary disease; CX=left circumflex artery; LMCA= left main coronary artery; NSTE-ACS=no ST-segment elevation acute coronary syndrome; NSTEMI=no ST-segment elevation myocardial Infarction; PAD= Peripheral arterial occlusive disease; RCA=right coronary artery; STEMI =ST-segment elevation myocardial infarction; UA=unstable angina.

**Table 2:** Procedural characteristics and concomitant antithrombotic medication use by oral P2Y_12_ inhibitor used.

	Clopidogrel (CLO) N= 138	Prasugrel (PRA) N=127	Ticagrelor (TICA) N=730	Eligible Patients N=995	P
Duration of cangrelor (minutes)*	151.4 (43.3)	140.2 (34.1)	150.0 (122.7)	148.8 (106.2)	0.6192
**< 2 hours**	6 (4.3%)	6 (4.7%)	23 (3.2%)	35 (3.5%)	0.6465
**2-4 hours**	128 (92.8%)	116 (91.3%)	689 (94.4%)	933 (93.8%)	
**> 4 hours**	3 (2.2%)	1 (0.8%)	10 (1.4%)	14 (1.4%)	
**Radial artery access**	123 (89.1%)	123 (96.9%)	680 (93.2%)	926 (93.1%)	0.0470
Duration of primary PCI (minutes)*	52.7 (35.9)	41.4 (26.2)	48.5 (37.3)	48.1 (36.0)	0.0363
**Any DES**	132 (95.7%)	126 (99.2%)	714 (97.8%)	972 (97.7%)	0.1777
**1 vessel with at least a DES**	111 (80.4%)	103 (81.1%)	585 (80.1%)	799 (80.3%)	0.9863
**2 vessels with at least a DES**	18 (13.0%)	20 (15.7%)	108 (14.8%)	146 (14.7%)	
**3 vessels with at least a DES**	3 (2.2%)	3 (2.4%)	21 (2.9%)	27 (2.7%)	
**Antithrombotic medication use administered in the 24 hours before or during PCI (w/wo other drugs)**					
**GPI peri-procedural/bailout use**	2 (1.4%)	3 (2.4%)	21 (2.9%)	26 (2.6%)	0.7215
**Duration of GPI (hours)^§^**	16.5 (0.0-33.3)	0.0 (0.0-0.0)	7.7 (0-21.3)	3.9 (0.0-21.3)	0.2325
**ASA**	138 (100.0%)	124 (97.6%)	729 (99.9%)	991 (99.6%)	0.0109
**UFH**	122 (88.4%)	105 (82.7%)	621 (85.1%)	848 (85.2%)	0.4197
**LMWH**	21 (15.2%)	5 (3.9%)	62 (8.5%)	88 (8.8%)	0.0052
**Fondaparinux**	5 (3.6%)	13 (10.2%)	41 (5.6%)	59 (5.9%)	0.0698
**DOACs**	22 (15.9%)	0 (0.0%)	0 (0.0%)	22 (2.2%)	<0.0001
**Vitamin K antagonists**	4 (2.9%)	0 (0.0%)	0 (0.0%)	4 (0.4%)	0.0006

**Note:** Values are mean (SD), median (Q1-Q3), n patients (%).

Fisher exact test for dicotomic variables.

* mean (SD); parametric ANOVA.

^§^ median (Q1-Q3); Kruskal-Wallis test.

Legend: ASA=acetylaalicylic acid; DES=drug-eluting stent; DOACs= oral direct factor Xa inhibitors and direct thrombin inhibitors; GPI=glycoprotein IIb/IIIa inhibitors; LMWH=low molecular weight heparin; PCI=percutaneous coronary intervention; UFH=unfractionated heparin; w/wo=with/without.

**Table 3:** Patients exposed to more than one oral P2Y_12_ inhibitor and reasons to switch.

	**Clopidogrel (CLO) N= (138)**	**Prasugrel (PRA) N= (127)**	**Ticagrelor (TICA) N= (730)**	**Eligible Patients N= (995)**
Patients exposed to more than one oral P2Y_12_ inhibitor*	6 (4.3%)	3 (2.4%)	51 (7.0%)	60 (6.0%)
**Reason to Switch**				
**New thrombotic event**	1 (0.7%)	0 (0.0%)	2 (0.3%)	3 (0.3%)
**Dyspnea**	0 (0.0%)	0 (0.0%)	9 (0.9%)	9 (0.9%)
**Physiscian's choice**	0 (0.0%)	0 (0.0%)	2 (0.2%)	2 (0.2%)
**Acute kidney failure**	0 (0.0%)	1 (0.3%)	0 (0.0%)	1 (0.1%)
**Anemia**	0 (0.0%)	0 (0.0%)	1 (0.1%)	1 (0.1%)
**Atrial Fibrillation**	0 (0.0%)	0 (0.0%)	1 (0.1%)	1 (0.1%)
**Bradyarthythmia and vomiting**	0 (0.0%)	0 (0.0%)	1 (0.1%)	1 (0.1%)
**Optimization of cardioactive therapy**	0 (0.0%)	0 (0.0%)	1 (0.1%)	1 (0.1%)
**Skin rash**	0 (0.0%)	0 (0.0%)	1 (0.1%)	1 (0.1%)
**Start novel NOACs therapy**	0 (0.0%)	0 (0.0%)	1 (0.1%)	1 (0.1%)
**Unknown**	5 (3.6%)	2 (1.6%)	32 (4.4%)	39 (3.9%)

**Note:** Values are n patients (%). * P = 0.0877.

Legend: NOACs=novel oral anticoagulants.

**Table 4:** Incidence of major adverse cardiac events (MACEs) within 48 (±24) hours and 30 (±3) days post-PCI.

**-**	**Clopidogrel (CLO) N=138**	**Prasugrel (PRA) N=127**	**Ticagrelor (TICA) N=730**	**Eligible Patients N=995**
Patients experiencing at least one MACE 48 hours*	3 (2.2%)	0 (0.0%)	7 (1.0%)	10 (1.0%)
**All-cause death**	1 (0.7%)	0 (0.0%)	3 (0.4%)	4 (0.4%)
**At least one MI (all types)**	2 (1.4%)	0 (0.0%)	3 (0.4%)	5 (0.5%)
***Type 1***	0 (0.0%)	0 (0.0%)	0 (0.0%)	0 (0.0%)
***Type 2***	1 (0.7%)	0 (0.0%)	0 (0.0%)	1 (0.1%)
***Type 4a***	1 (0.7%)	0 (0.0%)	3 (0.4%)	4 (0.4%)
***Type 4b***	0 (0.0%)	0 (0.0%)	0 (0.0%)	0 (0.0%)
**At least one IDR**	0 (0.0%)	0 (0.0%)	0 (0.0%)	0 (0.0%)
**At least one ST (all types)**	0 (0.0%)	0 (0.0%)	1 (0.1%)	1 (0.1%)
**Patients experiencing at least one MACE 30 days#**	6 (4.3%)	0 (0.0%)	8 (1.1%)	14 (1.4%)
**All-cause death**	3 (2.2%)	0 (0.0%)	3 (0.4%)	6 (0.6%)
**At least one MI (all types)**	3 (2.2%)	0 (0.0%)	4 (0.5%)	7 (0.7%)
***Type 1***	0 (0.0%)	0 (0.0%)	1 (0.1%)	1 (0.1%)
***Type 2***	2 (1.4%)	0 (0.0%)	0 (0.0%)	2 (0.2%)
***Type 4a***	1 (0.7%)	0 (0.0%)	3 (0.4%)	4 (0.4%)
***Type 4b***	0 (0.0%)	0 (0.0%)	0 (0.0%)	0 (0.0%)
**At least one IDR**	0 (0.0%)	0 (0.0%)	0 (0.0%)	0 (0.0%)
**At least one ST (all types)**	1 (0.7%)	0 (0.0%)	1 (0.1%)	2 (0.2%)

**Note:** Values are n patients (%). *p=0.1827; # p = 0.0113 Fisher exact test. **Legend**: MACE = major adverse cardiac events; IDR = ischemia-driven revascularization; MI = myocardial infarction; PCI = percutaneous coronary intervention; ST = stent thrombosis.

## Data Availability

The data and supportive information are available within the article.

## LIST OF ABBREVIATIONS

BARC: Bleeding academic research consortium
CAD: Coronary artery disease
CLO: Clopidogrel
IPTE: Intraprocedural thrombotic events
MACEs: Major adverse cardiac events
NACEs: Net adverse clinical events
PCI: Percutaneous coronary intervention
PRA: Prasugrel
TEAE: Treatment-emergent adverse events
TICA: Ticagrelor

## STANDARDS OF REPORTING

CONSORT guidelines were followed.
